# Integrative MRI and Genomics Analyses Prioritize *PACSIN1* as a Candidate Gene for Cerebellar Ataxia in Border Collies

**DOI:** 10.3390/ani16131987

**Published:** 2026-06-27

**Authors:** Ding-Jun Jin, Shuo-Chen Jiang, Jin-Xiu Li, Yan-Hu Liu, Bo-Wen Zhou, Ya-Ping Zhang

**Affiliations:** 1 State Key Laboratory of Genetic Evolution & Animal Models, Kunming Institute of Zoology, Chinese Academy of Sciences, Kunming 650223, China; 2 Kunming College of Life Science, University of Chinese Academy of Sciences, Kunming 650201, China; 3 Bio-X Center for Interdisciplinary Innovation and State Key Laboratory for Conservation and Utilization of Bio-Resources, Yunnan University, Kunming 650091, China; 4 KIZ-CUHK Joint Laboratory of Bioresources and Molecular Research in Common Diseases, Kunming Institute of Zoology, Chinese Academy of Sciences, Kunming 650223, China

**Keywords:** endophenotype, canine neurodegeneration, whole-genome sequencing, variant prioritization, cerebellar morphometry

## Abstract

Cerebellar ataxia is clinically and genetically heterogeneous, and candidate variant prioritization is particularly difficult when only a single affected animal is available. We combined structural MRI of one affected Border Collie and six controls with whole-genome sequencing of the affected dog and 31 comparison dogs. MRI revealed widened cerebellar folial spaces and reduced bilateral cerebellar gray matter volume, without global brain atrophy or spinal cord lesioning. Parallel heterozygous and recessive variant screens, together with identity-by-descent, runs-of-homozygosity, structural-variant, and repeat-expansion analyses, narrowed the candidates to six non-MODIFIER variants. Among these, a heterozygous *PACSIN1* splice-donor variant (chr12:3,985,222 T > G) was private to the affected dog in the analyzed cohort and affects a conserved residue in the F-BAR domain. No segregation, replication, or functional validation data are currently available. These results prioritize the *PACSIN1* variant as a testable candidate and demonstrate the utility of imaging-guided genomic prioritization in underpowered veterinary cohorts.

## Simple Summary

Cerebellar ataxia impairs balance and coordination, but identifying its genetic basis is difficult when only one affected animal is available. We combined clinical assessment, brain MRI, and whole-genome sequencing to investigate a Border Collie with early-onset progressive ataxia. MRI localized the main structural abnormality to the cerebellum. Genomic analyses prioritized a heterozygous splice-donor variant in *PACSIN1* as a candidate for further study. Given incomplete breeding records, *PACSIN1* remains a strong candidate rather than a confirmed causal gene. The study shows how imaging can guide genomic candidate prioritization in rare veterinary neurological cases.

## 1. Introduction

Cerebellar ataxia comprises a clinically and genetically heterogeneous group of neurodegenerative disorders characterized by impaired coordination, gait abnormalities, and progressive motor dysfunction [1]. Although numerous causal genes have been identified across species, a substantial proportion of cases remain genetically unresolved, reflecting both locus heterogeneity and limitations in study design. This challenge is particularly pronounced in rare or early-onset cases [2,3], where access to large cohorts for genetic mapping is inherently limited [4].

A further challenge is the imprecision of phenotype definition based solely on clinical observation [5,6,7,8], which can obscure genotype–phenotype relationships. Increasing evidence suggests that deep phenotyping, particularly using quantitative imaging, can refine disease classification and improve the interpretability of genetic data [9,10]. However, integrative frameworks that systematically combine imaging-defined phenotypes with genomic data for variant prioritization remain underdeveloped [11,12]. This gap is especially relevant in structured populations, such as domestic animals, where reduced genetic diversity and well-defined pedigrees provide unique opportunities for genetic discovery, even with limited sample sizes [13]. The domestic dog represents a powerful model for studying inherited neurological disorders due to its population structure [14], breed-specific disease enrichment, and increasing availability of large-scale genomic resources [15,16]. In particular, closed or semi-closed breeding populations enable efficient filtering of high-risk variants when combined with appropriate phenotypic constraints [3,17].

Advances in canine genomics have identified over 400 Mendelian disease variants across breeds [18], and large-scale screening shows that approximately 57% of dogs carry at least one known disease-associated allele [19]. However, the extended linkage disequilibrium and population structure that facilitate trait mapping also increase the likelihood that benign alleles appear private in small breed cohorts. Resources such as the Dog10K consortium, which has assembled over 2000 canid genomes spanning 321 breeds [20], can help distinguish common polymorphisms from rare variants, although genotype–phenotype associations still require independent validation [21].

Here, we investigated a Border Collie with early-onset progressive ataxia using clinical assessment, structural MRI, and whole-genome sequencing. MRI refined the neuroanatomical phenotype, after which we evaluated heterozygous and recessive SNPs, relatedness, runs of homozygosity, structural variants, repeat-expansion signals, and predicted splice effects. We prioritized *PACSIN1*, which contains a candidate site predicted to disrupt a splice donor and encodes a protein with established neuronal functions. Our aim was to generate a testable candidate hypothesis, not to establish causality from a single affected dog.

## 2. Materials and Methods

### 2.1. Animals and Study Design

This study investigated a closed population of 32 Border Collies (Table S1), in which one individual exhibited a severe cerebellar ataxia phenotype. The affected dog developed progressive hindlimb motor abnormalities between 6 and 8 months of age, characterized by gait instability and impaired coordination. Following clinical neurological evaluation by a licensed veterinarian, cerebellar ataxia was suspected, and the dog subsequently underwent MRI examination at the Kunming Institute of Zoology.

Six age-matched Border Collies without neurological or behavioral abnormalities, as confirmed by veterinary assessment, were included as controls and underwent MRI scanning at the Kunming Institute of Zoology. None of the dogs received any treatment prior to imaging. Because the phenotype was rare and only one affected dog was available, the MRI component was designed as an exploratory single-case phenotyping analysis rather than a conventional case–control study powered for population-level statistical inference.

Peripheral blood or saliva samples were collected from all available individuals in the cohort, and genomic DNA was extracted for sequencing. Whole-genome sequencing was performed on the NovaSeq 6000 platform (Illumina, San Diego, CA, USA) using a paired-end 150 bp (PE150) strategy. The affected individual (DZ) was sequenced to an average depth of 20×, while the remaining 31 phenotypically normal dogs were sequenced at an average depth of approximately 10×.

### 2.2. MRI Acquisition

Before each MRI scanning session, anesthesia was induced with an intramuscular injection of Zoletil 50 (Virbac S.A., Carros, France) at 2 mg/kg and Dexdomitor (Orion Corporation, Espoo, Finland) at 0.01 mg/kg. General anesthesia was then achieved with propofol to effect (2–3 mg/kg), followed by intubation. Anesthesia was maintained with 1.5% isoflurane using the pressure-controlled ventilation (PCV) mode, with ventilator parameters set to 12 breaths/min and an inspiratory pressure of 12 mmH_2_O. The animal was positioned supinely within the MRI scanner, with its head secured in a knee coil using circumferential sponge padding to minimize respiratory-induced motion artifacts.

3D T1-weighted and T2-weighted structural images were acquired in the affected dog (DZ) and in the control dogs using the same MRI system and coil, but not during the same imaging session. The six control dogs were scanned using an identical acquisition protocol, and the affected dog was scanned using a closely matched clinical protocol with differences in imaging orientation, TR/TE, and number of slices. Identical acquisition parameters could not be applied because the affected dog was scanned as part of a clinical/diagnostic evaluation, whereas the control dogs were selected retrospectively from the available MRI dataset. Because all data were acquired using 3D brain imaging sequences without inter-slice gaps, slice orientation and number did not affect image quality. To reduce processing-related variability, all T1-weighted images were processed using the same procedures. Nevertheless, potential acquisition-related effects on morphometric estimates cannot be fully excluded.

For the affected dog, 3D T1-weighted brain images were acquired using a Fast Spoiled Gradient Echo Imaging (GRE-FSP) sequence with the following parameters: slice thickness = 0.5 mm, number of slices = 110, sagittal orientation, averages = 3, TR = 13 ms, TE = 5.6 ms and TI = 880 ms. 3D T2-weighted images were acquired using a MATRIX sequence with the following parameters: slice thickness = 0.5 mm, number of slices = 110, sagittal orientation, averages = 2, TR = 3400 ms, and TE = 396.5 ms. T2-weighted spine images were acquired with the following parameters: slice thickness = 2 mm, number of slices = 15, sagittal orientation, averages = 2, TR = 1912 ms, and TE = 96.66 ms.

For the six control dogs, 3D T1-weighted images were acquired using the same GRE-FSP sequence with the following parameters: slice thickness = 0.5 mm, number of slices = 150, transverse orientation, averages = 3, TR = 11.7 ms, TE = 3.9 ms and TI = 960 ms. 3D T2-weighted images were acquired using the same MATRIX sequence with the following parameters: slice thickness = 0.5 mm, number of slices = 150, coronal orientation, averages = 2, TR = 2300 ms, and TE = 338.9 ms. T1-weighted images were used for volumetric analysis, whereas T2-weighted images were used for visual assessment of brain and spinal cord abnormalities.

### 2.3. MRI Data Preprocessing

T1-weighted images were processed using ANTs, AFNI, and FSL [22,23,24]. Briefly, images underwent N4 bias field correction and skull stripping, followed by registration to the Corndog canine brain template. A study-specific template was then generated from all skull-stripped T1-weighted images using antsMultivariateTemplateConstruction2. Label tissue priors derived from the Corndog template were mapped to the study-specific template and used to guide tissue segmentation into gray matter, white matter, and cerebrospinal fluid with Atropos [22,25]. Total intracranial volume (TIV) was calculated as the sum of gray matter volume (GMV), white matter volume (WMV), and cerebrospinal fluid volume (CSFV) [26].

### 2.4. Statistical Analysis of MRI Data

Comparisons between the single affected dog and the control group were performed within a single-case inferential framework based on the t-distribution. Specifically, following the conventional pooled-variance approach described for single-subject morphometric analyses, the affected dog’s value was compared with the control sample while treating the control mean and standard deviation as sample statistics rather than population parameters. To account for inter-individual differences in head size, this framework was implemented as an ANCOVA/general linear model with TIV entered as a covariate [27,28]. The significance level was set at *p* < 0.05.

### 2.5. Sequence Alignment and Variant Calling

Raw sequencing reads were first processed using fastp (v0.23.2) for quality control with the following parameters: -q 5, -u 50, -n 15, and -l 150 [29]. The filtered reads were then aligned to the canine reference genome (UU_Cfam_GSD_1.0) using BWA-MEM (v0.7.17) with default settings [30].

Subsequently, duplicate reads were marked using MarkDuplicates, and base quality score recalibration (BQSR) was performed using BaseRecalibrator implemented in GATK (v4.2.6.300). Variant calling was conducted using the HaplotypeCaller in GVCF mode, followed by joint genotyping across all samples using GenotypeGVCFs.

The initial variant set was first refined using Variant Quality Score Recalibration (VQSR) [31,32,33,34]. In addition, a hard filtering step was applied with support from the Dog10K dataset (prior = 15.0) to obtain a high-confidence variant set. The filtering criteria were as follows [35]:

SNPs: QD < 2.0, QUAL < 50.0, MQ < 40.0, FS > 60.0, SOR > 3.0, MQRankSum < −12.5

Indels: QD < 2.0, FS > 200.0, SOR > 10.0, InbreedingCoeff < −0.8

A closed Border Collie population was established as an internal reference panel to facilitate variant filtering. Variant calling files were first normalized using bcftools (v1.21) [36], after which private variants in the affected individual were identified. Specifically, we extracted sites that were exclusively present in the affected dog (allele count AC = 1, allele number AN = 64) and had no missing genotypes across the cohort.

To further prioritize potentially causal variants, we removed known polymorphic sites by comparison against the public Dog10K database [20,37], retaining putative novel variants. Subsequently, stringent quality control filters were applied [35], including genotype quality (GQ ≥ 40), sequencing depth (DP ≥ 20), and allelic balance (0.25 ≤ AB ≤ 0.75), resulting in a set of high-confidence candidate variants for downstream analysis.

### 2.6. Structural Variant Analysis

Structural variants were called with Manta (v1.6.0) [38] and filtered using cohort and Dog10K interval comparisons [39]. Canonical structural variants were classified as insertion, breakend, deletion, duplication, or inversion, and record-level PASS status and overlap with the six prioritized SNP candidate genes were assessed.

### 2.7. Population Genetic Analysis

Population structure was evaluated by principal component analysis of genome-wide autosomal SNPs. Pairwise identity-by-descent was estimated after restricting variants to a minor-allele frequency of at least 0.05 and applying linkage-disequilibrium pruning; the remaining SNPs were retained for identity-by-descent (IBD) analysis. Pairwise PI_HAT values were used to describe genetic relatedness but not to assign pedigree identities.

Runs of homozygosity were identified across the autosomes using PLINK (v1.90b) with a 200 kb minimum length, a 100 SNP sliding window, and a heterozygous call allowance of 5 per window. FROH was summarized per dog, and candidate variants were intersected with ROH at 5, 10, and 20 Mb thresholds.

### 2.8. Variant Annotation, Splice Prediction, Repeat-Expansion Screening, and Functional Enrichment

Candidate variants were annotated using the Ensembl Variant Effect Predictor (VEP) [40]. Variants were prioritized based on their predicted functional impact (IMPACT), with particular emphasis on those classified as moderate (e.g., missense_variant) and high-impact (e.g., splice_donor_variant).

Predicted splice effects of the six non-MODIFIER heterozygous candidates were evaluated using SpliceAI (v1.3.1) (https://github.com/Illumina/SpliceAI, accessed on 24 June 2026). Delta scores for acceptor gain, acceptor loss, donor gain, and donor loss were recorded.

Repeat-associated signals were evaluated with ExpansionHunterDenovo (v0.9.0) (https://github.com/Illumina/ExpansionHunterDenovo, accessed on 24 June 2026). Anchored in-repeat profiles were generated for DZ and the 31 comparison dogs. Official locus-level case–control outlier analyses were performed with DZ as the case. A top-case *z*-score of at least 10 was prespecified as the threshold for a strong outlier signal.

Unique canine genes annotated by VEP were mapped to their human orthologues. Only one-to-one orthologues were retained. Gene Ontology and KEGG over-representation analyses were performed using clusterProfiler (v4.14.6) [41]. *p*-values were adjusted using the Benjamini–Hochberg method, and adjusted *p*-values below 0.05 were considered statistically significant.

### 2.9. Protein Domain and Evolutionary Conservation Analysis

Protein functional domains of candidate genes were annotated using InterProScan and the UniProt database [42,43]. Candidate variants were categorized according to their predicted functional IMPACT, and their positions within protein domains were visualized using ggplot2 (v4.0.1) [44].

To assess evolutionary conservation, canonical protein isoforms from representative species, including human, dog, spotted hyena, cattle, mouse, and rat, were analyzed. Homologous sequences were identified using the National Center for Biotechnology Information (NCBI) BLASTp (v2.17.0), followed by multiple sequence alignment (MSA) and visualization using DNAMAN (v9.0).

## 3. Results

### 3.1. Clinical Presentation of the Affected Dog

**Figure 1:**
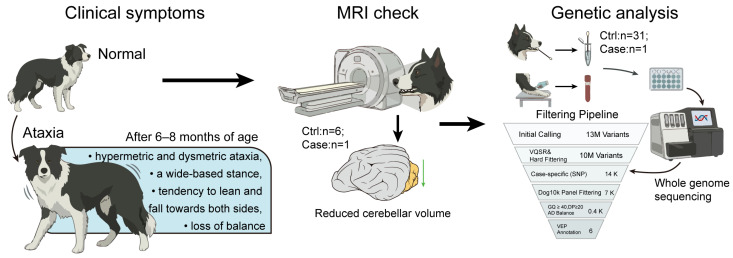
Schematic workflow of cerebellar ataxia in Border Collies: from clinical phenotyping to genetic analysis. Clinical symptom assessment. Comparison between the affected dog (Ataxia) and the normal control is shown. The affected dog exhibits typical ataxia symptoms after 6–8 months of age, including hypermetric and dysmetric gait, wide-based stance, leaning, and loss of balance. MRI examination. MRI scans were performed on the case (*n* = 1) and controls (*n* = 6). Results indicate a significant reduction in cerebellar volume in the affected individual. Genetic analysis and variant filtering pipeline. Whole-genome sequencing (WGS) was conducted on the case (*n* = 1) and controls (*n* = 31). The filtering funnel illustrates the bioinformatic pipeline, including VQSR, case-specific SNP filtering, Dog10K panel filtering, and VEP functional annotation, leading to the identification of candidate variants. Arrows indicate the sequential analytical workflow from clinical phenotyping to MRI assessment and genetic analysis.

**Figure 2:**
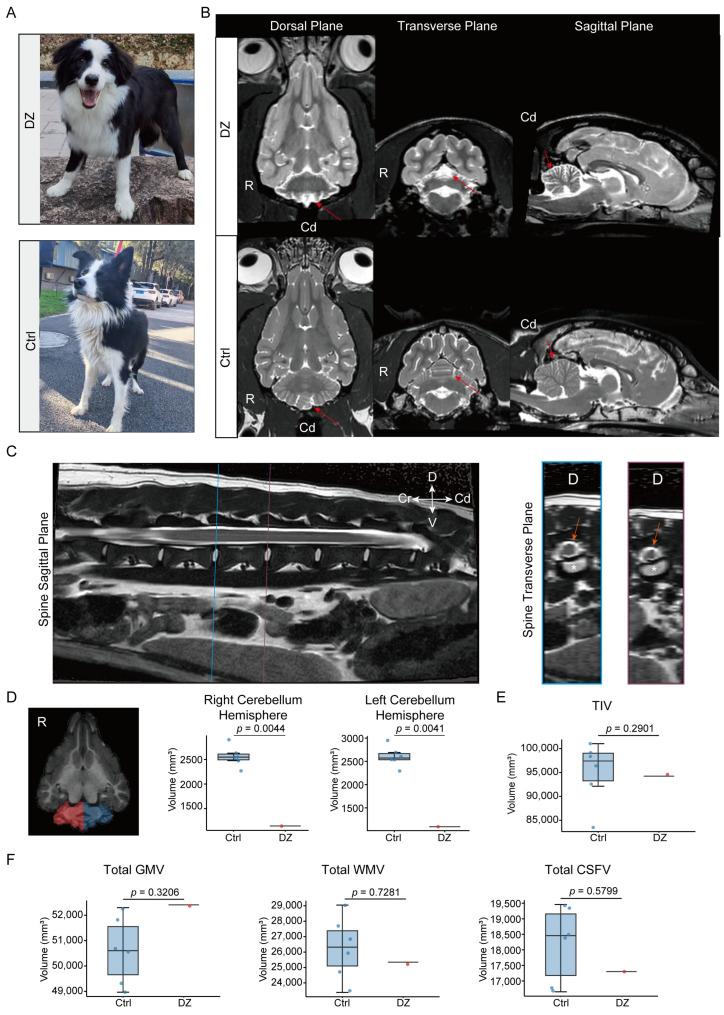
Brain and spinal MRI images and volumetric analysis. (**A**) Representative clinical images of the affected dog (DZ) and a control dog (Ctrl). DZ showed a wide-based stance. (**B**) Representative T2-weighted brain images of DZ and a control dog (Ctrl) in dorsal, transverse, and sagittal planes. Compared with Ctrl, DZ showed widened cerebellar folial spaces and increased pericerebellar cerebrospinal fluid spaces. Red arrows indicate pericerebellar cerebrospinal fluid spaces, which were enlarged in DZ compared with Ctrl. (**C**) T2-weighted spinal images of DZ in sagittal and transverse planes. The blue and brown vertical lines in the sagittal spinal image indicate the anatomical levels corresponding to the transverse images shown on the right. Asterisks indicate intervertebral discs. Orange arrows indicate the spinal cord in transverse T2-weighted images. No obvious abnormal spinal cord signal, compression, or apparent intervertebral disc degeneration was observed. (**D**) T1-weighted image with template-based cerebellar segmentation and quantitative comparison of cerebellar gray matter volume. The right and left cerebellar gray matter regions are shown in red and blue, respectively. (**E**) Comparison of total intracranial volume (TIV) between control dogs and DZ. (**F**) Whole-brain volumetric measures, including total gray matter volume (GMV), total white matter volume (WMV), and cerebrospinal fluid volume (CSFV). Boxplots show the median, interquartile range, and range of the control group; blue points indicate individual control dogs. DZ is shown as a single red point. R, right; Cd, caudal; Cr, cranial; D, dorsal; V, ventral.

DZ began exhibiting abnormal hindlimb movements at 6–8 months of age, with rapid disease progression thereafter (Figure 1). The primary clinical manifestations included unstable gait, aberrant hindlimb motions, and diminished motor coordination. Gait observation showed that DZ had broad-based stance/gait and dysmetria, consistent with clinical characteristics of cerebellar ataxia (Figure 2A, Videos S1 and S2). To further evaluate potential structural abnormalities in the cerebellum and rule out other neurological pathologies, magnetic resonance imaging (MRI) and genetic analysis were performed on the subject.

### 3.2. MRI Analysis Identified the Cerebellum as the Primary Affected Brain Region

MRI examination provided anatomical evidence of cerebellar involvement in DZ. On T2-weighted brain images, DZ showed widening of the cerebellar folial spaces and increased pericerebellar cerebrospinal fluid spaces, most clearly visible in the sagittal plane and also appreciable on the dorsal and transverse planes (Figure 2B). These findings were consistent with cerebellar atrophy and matched the observed clinical signs of cerebellar ataxia. In contrast, T2-weighted spinal imaging did not reveal any obvious abnormal spinal cord signal or apparent intervertebral disc degeneration at the examined levels (Figure 2C). Together, the brain and spinal T2w MRI findings indicated that the structural abnormality was primarily localized to the cerebellum, without clear evidence of a concurrent spinal cord lesion.

T1-weighted images were used for template-based tissue segmentation and volumetric analysis, including cerebellar gray matter, total gray matter, total white matter, and cerebrospinal fluid. The volumetric analysis showed that cerebellar gray matter volume was reduced in both cerebellar hemispheric regions of DZ compared with control dogs, with significant differences in the right and left cerebellar gray matter volumes (right: *p* = 0.0044 and left: *p* = 0.0041; Figure 2D). TIV did not differ significantly between DZ and control dogs (*p* = 0.2901; Figure 2E). In a general linear model with TIV included as a covariate, total GMV, WMV, and CSFV were not significantly different between DZ and control dogs (*p* = 0.3206, 0.7281, and 0.5799, respectively; Figure 2F). Together, these findings indicate that the structural abnormality was mainly characterized by reduced bilateral cerebellar gray matter volume, without clear evidence of a whole-brain volumetric change.

### 3.3. Genetic Analysis Delineates the Familial Relationships of the Affected Dog

**Figure 3:**
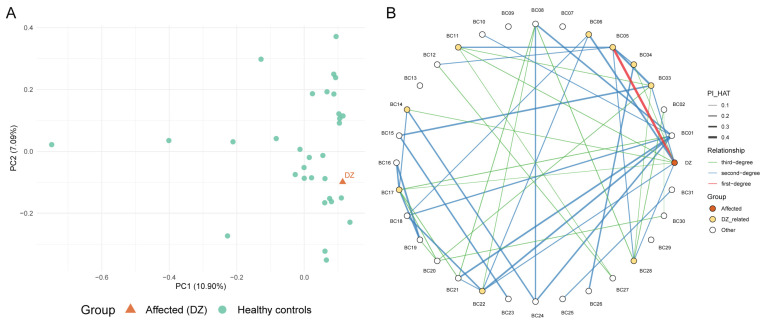
Population structure and pairwise genetic relatedness in the cohort. (**A**) Principal component analysis of genome-wide genotypes from DZ and healthy Border Collies. DZ is shown as an orange triangle and healthy controls as green circles. (**B**) Identity-by-descent (IBD) network based on linkage disequilibrium-pruned autosomal SNPs. Nodes represent individual dogs, with DZ shown in orange, dogs genetically related to DZ at the displayed threshold shown in yellow, and all other dogs shown in white. Edge colors indicate the inferred degree of relationship, and edge width is proportional to PI_HAT.

Whole-genome sequencing of DZ and the closed cohort initially identified 13,052,448 variants, including 7,388,551 SNPs and 5,663,897 InDels. Following the strict heterozygous filtering strategy described in the Section 2, a total of 362 PASS SNPs were retained for which DZ carried a heterozygous alternate allele after cohort-genotype, external-variation, genotype-quality, depth, and allelic-balance filtering (Figure 3, Table S2). A parallel recessive screen retained 597 PASS SNPs for which DZ was homozygous alternate and no other dog was homozygous alternate (Table S3). The two screens were carried forward as complementary candidate sets rather than evidence for a predetermined mode of inheritance.

Principal component analysis placed DZ within the genetic variation of the sampled Border Collie cohort rather than as a distinct population outlier (Figure 3A). IBD analysis based on 98,612 LD-pruned SNPs nevertheless demonstrated substantial within-cohort relatedness (Figure 3B and Table S4). DZ showed the highest pairwise sharing with dog BC05 (PI_HAT of 0.4351), followed by dog BC06 (PI_HAT of 0.3211), and additional dogs fell within ranges compatible with more distant relationships (Figure S1). These estimates confirmed cryptic or close relatedness but could not identify parents, littermates, or the direction of inheritance in the absence of recorded pedigree information.

### 3.4. Genome-Wide Candidate Variant Landscape of the Affected Dog

**Figure 4:**
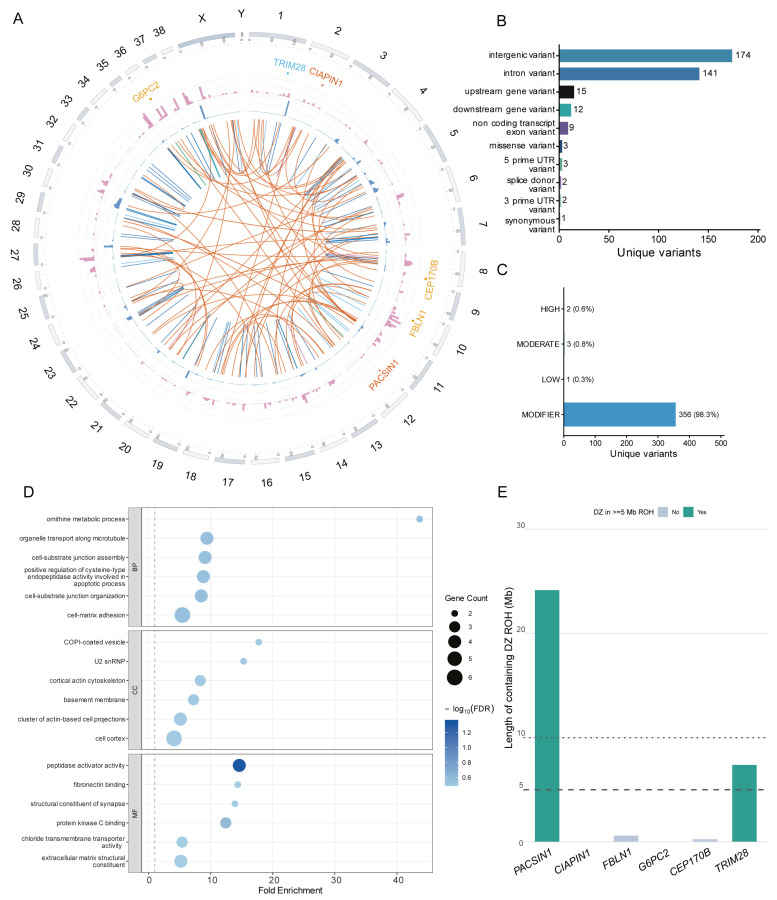
Genome-wide landscape of the affected dog. (**A**) Circos plot illustrating the genomic landscape of DZ-specific variants. Canine chromosome ideograms form the outer ring. Concentric tracks from inside to outside display: DZ-specific structural variants (SVs), DZ-specific SNPs under de novo/dominant filtering, and DZ-specific SNPs under recessive filtering. Genes annotated with predicted impacts derived from the de novo/dominant filtering category are labeled at their corresponding genomic positions. (**B**) Classification of candidate variants by Variant Effect Predictor (VEP) impact category. (**C**) Distribution of candidate variants by predicted molecular consequence and genomic annotation. (**D**) Gene Ontology enrichment analysis of genes associated with the candidate variants, separated into biological process (BP), cellular component (CC), and molecular function (MF). The X-axis shows fold enrichment, point size represents gene count, and color represents statistical significance after multiple-testing correction. (**E**) Lengths of ROH segments containing the prioritized variants in DZ. Bar color indicates whether a variant is located within a long ROH, and horizontal reference lines indicate the ROH-length thresholds used in the analysis. The dashed horizontal reference lines mark 5 Mb and 10 Mb ROH thresholds.

When each of the 362 heterozygous candidate sites was assigned its highest VEP impact (Figure 4A and Figure S2), two were classified as HIGH, three as MODERATE, one as LOW, and 356 as MODIFIER (Figure 4B,C). The six non-MODIFIER sites comprised splice-donor variants in *PACSIN1* and *CIAPIN1*; missense variants in *FBLN1*, *G6PC2*, and *CEP170B*; and a synonymous variant in *TRIM28*. No non-reference genotype was called at these sites in the 31 comparison dogs. However, median site-level depth was only approximately one to three reads; complete allele absence therefore cannot be established.

Gene Ontology (GO) enrichment of the VEP-annotated genes returned neuronal and synaptic terms, including GABAergic synapse and COPI-coated vesicle, the latter containing *PACSIN1* (Figure 4D). Only peptidase activator activity, represented in part by *FBLN1*, was nominally significant (adjusted *p* = 0.043; Table S5). No KEGG pathway reached significance (Figure S3 and Table S6). Enrichment was therefore not used to rank the six prioritized candidates. DZ had the highest FROH from long ROH in the cohort (≥5 Mb: 0.2076, 5.10-fold above the comparison-dog mean) and ranked second in overall FROH (0.3357). *PACSIN1* and TRIM28 fell within DZ ROH ≥ 5 Mb, and *PACSIN1* lay within an ROH ≥ 10 Mb (Figure 4E and Figure S4). These findings indicate extensive recent shared ancestry but do not establish a causal link between the ROH, the candidate alleles, and ataxia.

### 3.5. PACSIN1 Is Prioritized as a Biologically Plausible Candidate

**Figure 5:**
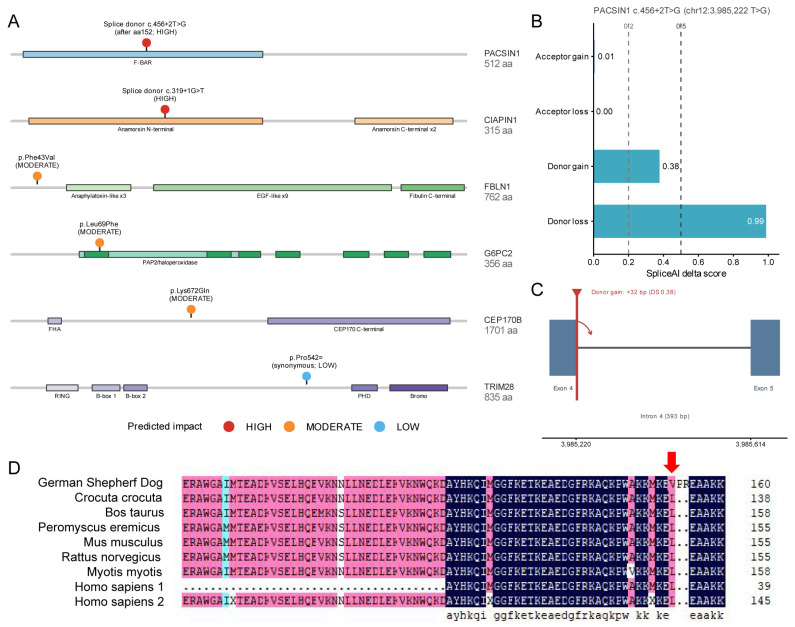
Functional interpretation of candidate gene variants. (**A**) Protein domain architectures of the candidate genes, with the positions and predicted consequences of the candidate variants indicated above each protein. Protein lengths are shown on the right, and variant markers are colored according to predicted impact. (**B**) Predictions for the *PACSIN1* splice-donor variant, showing delta scores for acceptor gain, acceptor loss, donor gain, and donor loss. Dashed vertical lines indicate the reference thresholds used to interpret the predictions. (**C**) Schematic representation of the *PACSIN1* exon–intron region surrounding the candidate variant. The native splice-donor site and the predicted cryptic donor site are indicated, with genomic coordinates shown below. (**D**) Multiple sequence alignment (MSA) of the *PACSIN1* protein across representative species, including human (*Homo sapiens*), dog (*Canis lupus familiaris*), cattle (*Bos taurus*), rat (*Rattus norvegicus*), and mouse (*Mus musculus*). The sequences correspond to canonical protein isoforms. The red arrow indicates the amino acid position affected by the *PACSIN1* variant.

Among the six non-MODIFIER candidates, *PACSIN1* was prioritized for detailed characterization on the basis of its predicted splice-donor disruption and its neuronal function. The prioritized *PACSIN1* signal arose from one heterozygous genomic SNP, chr12:3,985,222 T > G (Figure 5A and Figure S5). VEP assigned a canonical splice-donor consequence (c.456+2 T > G), whereas an alternative transcript produced a missense annotation (c.458 T > G; p.Val153Gly). These are transcript-dependent annotations of the same allele, not two independent variants or a dual hit. SpliceAI predicted strong donor loss for *PACSIN1* (delta score = 0.99) with a weaker donor gain signal (delta score = 0.38) (Figure 5B,C), but the *CIAPIN1* splice-donor candidate received an identical score, indicating that splice prediction alone did not distinguish between these two candidates (Table S7). Additionally, no repeat locus or motif reached the prespecified outlier threshold (Table S8).

*PACSIN1* was prioritized because of the predicted splice effect and its established neuronal roles in membrane remodeling and synaptic-vesicle trafficking. The data do not establish transcript disruption, protein loss, haploinsufficiency, or a dominant-negative mechanism. The five remaining non-MODIFIER variants reside in genes with diverse cellular roles, including centrosomal organization (*CEP170B*), extracellular matrix biology (*FBLN1*), and transcriptional regulation (*TRIM28*) (Figure 5A). None carried a second variant in the same gene, and no functional or segregation evidence supports a modifier role.

To further evaluate the potential functional impact of the *PACSIN1* variant, we performed cross-species multiple sequence alignment (MSA) of the affected region (Figure 5D). The analysis demonstrated strong evolutionary conservation of the amino acid sequence encompassing the V153G substitution across diverse mammalian species, including dog (*Canis lupus familiaris*, *German Shepherd*), spotted hyena (*Crocuta crocuta*), cattle (*Bos taurus*), mouse (*Mus musculus*), rat (*Rattus norvegicus*), and human (*Homo sapiens*). This conservation is consistent with functional constraint on the F-BAR domain but does not independently demonstrate pathogenicity of the candidate allele.

## 4. Discussion

In this study, we performed a comprehensive analysis of a closed Border Collie population in which a single individual presented with cerebellar ataxia. The affected dog exhibited early-onset clinical signs, including a wide-based gait and dysmetria. Combined with single-case quantitative MRI analysis, we identified a significant reduction in bilateral cerebellar gray matter volume. Subsequent whole-genome sequencing enabled the prioritization of a candidate variant in *PACSIN1* as a potential contributor to the rapid disease progression observed in this case.

MRI played a central role in linking the clinical presentation to a defined neuroanatomical phenotype in this study. The affected dog showed widened cerebellar folial spaces and increased pericerebellar cerebrospinal fluid spaces on T2-weighted brain MRI, together with reduced bilateral cerebellar gray matter volume on T1-based morphometric analysis. In contrast, spinal MRI did not reveal obvious spinal cord signal abnormality, compression, or apparent intervertebral disc degeneration. These findings supported cerebellar involvement as the major structural correlate of the observed wide-based gait and dysmetria, rather than a primary spinal cord lesion. This imaging-based interpretation is consistent with previous reports of canine cerebellar ataxia in which MRI or neuroanatomical assessment helped localize the disease process to the cerebellum and supported subsequent genotype–phenotype interpretation.

Importantly, our study extended this qualitative imaging approach by combining visual assessment of T2-weighted abnormalities with T1-based template segmentation and quantitative volumetric analysis. This approach provided an objective estimate of bilateral cerebellar gray matter reduction and supported the definition of a cerebellar imaging-derived endophenotype [17,45,46,47]. In the present study, the observed cerebellar atrophy aligns well with the clinical manifestations of ataxia [48], supporting cerebellar involvement as a major neuroanatomical correlate of the affected dog’s phenotype. MRI therefore served not only to discover structural cerebellar abnormalities, but also to define an imaging-derived endophenotype that helped constrain interpretation of the genomic findings. This supports the broader value of imaging-defined phenotypes in the investigation of rare neurological disorders, particularly when genetic analyses are limited by small or underpowered cohorts [9,49].

Most previously reported cases of canine cerebellar cortical degeneration (CCD/CA) were characterized by an autosomal recessive mode of inheritance [7,17,50], although this predominance may partly reflect ascertainment bias—recessive alleles are efficiently detected by homozygosity mapping in structured breeds, whereas dominant or de novo mutations are harder to identify without trio sequencing. In the present study, the prioritized *PACSIN1* variant is heterozygous. Despite lying within a ≥10 Mb run of homozygosity in DZ, it was not detected in the most closely related cohort members (PI_HAT up to 0.4351), a pattern consistent with a recent mutational origin on a shared haplotype background; however, formal confirmation of de novo status would require trio data. Comparison with over 2000 genomes from the Dog10K dataset further supports the rarity of these variants [36,37,39]. Because parental genotypes were not available (Table S4), segregation analysis could not be performed, and the inheritance pattern remains unresolved. These findings therefore support variant prioritization rather than causal assignment and highlight the utility of combining WGS with a structured population to investigate sporadic neurological presentations.

Among the prioritized candidates, *PACSIN1* represents the most biologically plausible gene. It encodes Syndapin I, a protein essential for synaptic vesicle endocytosis and membrane remodeling [51,52,53]. Cross-species multiple sequence alignment demonstrates that the affected residue is highly conserved across vertebrates, and the variant localizes to the F-BAR domain, a key structural element responsible for membrane curvature sensing and stabilization [52]. If the variant alters *PACSIN1* splicing or protein function, this non-conservative substitution could affect the structural integrity of the F-BAR domain. Functional annotation indicates that this single-nucleotide variant may affect both the encoded amino acid sequence and splice-donor recognition across multiple transcripts [54]. Computational predictions alone, however, cannot distinguish whether such a dual annotation reflects true transcript-level disruption, and no RNA or protein data are currently available to test these possibilities. Experimental determination of the transcript and protein consequences is required before inferring haploinsufficiency, a dominant-negative mechanism, or impairment of synaptic vesicle recycling.

To place our findings in the context of existing studies, we surveyed candidate genes reported in canine cerebellar ataxia or ataxia-associated disorders across more than 26 dog breeds. Notably, the genes implicated in different breeds are largely non-overlapping, involving both monogenic and, in some cases, digenic inheritance patterns [3,55,56,57]. This observation highlights that canine ataxia exhibits marked genetic heterogeneity but converges on a limited set of neurodegenerative pathways.

The present study provides an imaging-informed genomic prioritization framework that is well suited to rare neurological presentations in structured populations, but several constraints should be noted. First, the study was based on a single affected dog without an independent replication cohort; the imaging findings are therefore exploratory and the genomic findings are candidate-generating rather than conclusive. Second, MRI acquisition parameters differed between DZ and the control dogs because of the clinical origin of the index scan, and parental genotypes were unavailable, preventing formal segregation analysis. Third, VEP and SpliceAI provide computational predictions that require transcript- and protein-level validation, and short-read sequencing has incomplete sensitivity for complex structural or repeat-mediated variation. The *PACSIN1* result should therefore be considered a prioritized hypothesis requiring segregation, replication, and functional validation.

## 5. Conclusions

Clinical assessment and structural MRI localized the primary abnormality in DZ to the cerebellum, revealing reduced bilateral cerebellar gray matter volume through an exploratory single-case comparison. Subsequent genome-wide analyses prioritized candidate variants by integrating parallel heterozygous and recessive SNP screens with relatedness, ROH, splice-prediction, structural-variant, and repeat-expansion evaluations. Our leading hypothesis points to a heterozygous variant in *PACSIN1* (chr12:3,985,222 T > G) predicted to disrupt donor splicing, an allele that remained private to DZ within the analyzed cohort. As no segregation, replication, transcript, or protein-level evidence is currently available, a definitive causal relationship cannot yet be established. Ultimately, the principal contribution of this study is the establishment of an imaging-informed genomic prioritization framework, yielding a defined set of testable candidates for future investigation in Border Collies presenting with comparable neurological phenotypes.

## Data Availability

The sequencing data generated during this study are available in the Genome Sequence Archive (GSA, https://ngdc.cncb.ac.cn/gsa/, accessed on 24 June 2026) under accession number CRA012878.

## Supplementary Materials

The following supporting information can be downloaded at https://www.mdpi.com/article/10.3390/ani16131987/s1: Video S1: Indoor locomotion of DZ; Video S2: Outdoor locomotion of DZ; Figure S1: Pairwise genomic relatedness in the Border Collie cohort; Figure S2: Comparison of de novo/dominant and recessive variant filtering strategies; Figure S3: KEGG pathway enrichment of VEP-annotated genes; Figure S4: Genome-wide runs of homozygosity burden in DZ and comparison dogs; Figure S5: Quality evidence for DZ-specific heterozygous candidate variants; Table S1: Sample information; Table S2: Private coding variants retained under de novo/dominant filtering; Table S3: Private coding variants retained under recessive filtering; Table S4: Results of the genetic analysis of the affected dog’s lineage; Table S5: GO enrichment analysis results. Table S6: KEGG enrichment analysis results; Table S7: SpliceAI predictions for prioritized variants; Table S8: Locus-level novel expansions of short tandem repeat (STRs) results.
