# Supplementary material for: Integrative MRI and Genomics Analyses Prioritize PACSIN1 as a Candidate Gene for Cerebellar Ataxia in Border Collies
Source: Animals (Basel). 2026 Jun 27;16(13):1987. doi: 10.3390/ani16131987 (PMC13359611; doi:10.3390/ani16131987)
Supplement: Supplementary file 1 [file animals-16-01987-s001.zip › Supplementary_Materials.pdf]

Supplementary Materials

Supplementary Figures

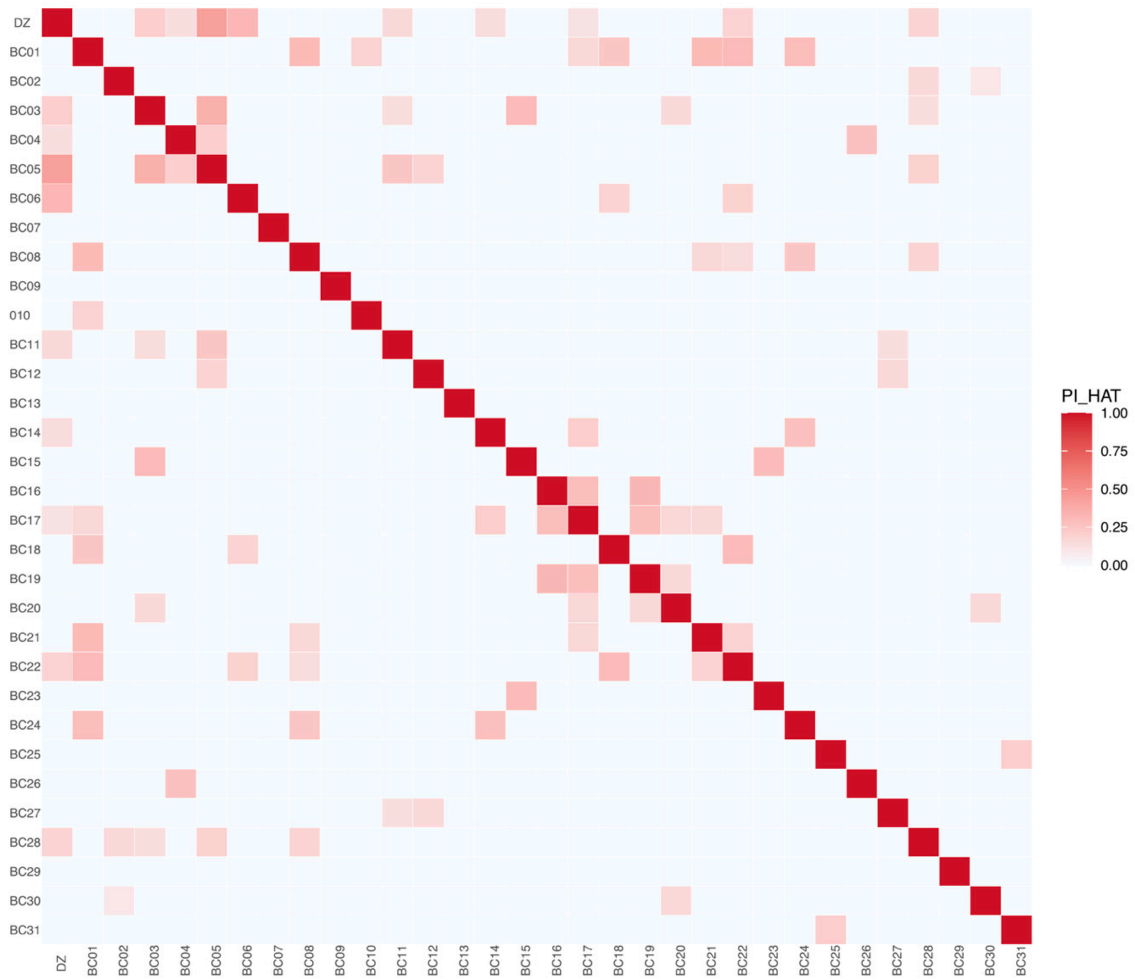

Figure S1. Pairwise genomic relatedness in the Border Collie cohort. The heatmap shows PLINK PI_HAT estimates calculated from 98,612 autosomal SNPs after masking genotypes with read depth < 3, applying a minor-allele-frequency threshold of 0.05 and a variant missingness threshold of 0.10, and linkage-disequilibrium pruning. Diagonal values equal 1, whereas darker off-diagonal cells indicate greater estimated identity-by-descent sharing. DZ showed the greatest relatedness to dog BC05 (PI_HAT = 0.4351), followed by dog BC06 (PI_HAT = 0.3211), with additional relationships in the approximate second- and third-degree ranges. These data demonstrate substantial cohort relatedness but, without recorded pedigree metadata, do not establish specific parent-offspring or littermate relationships.

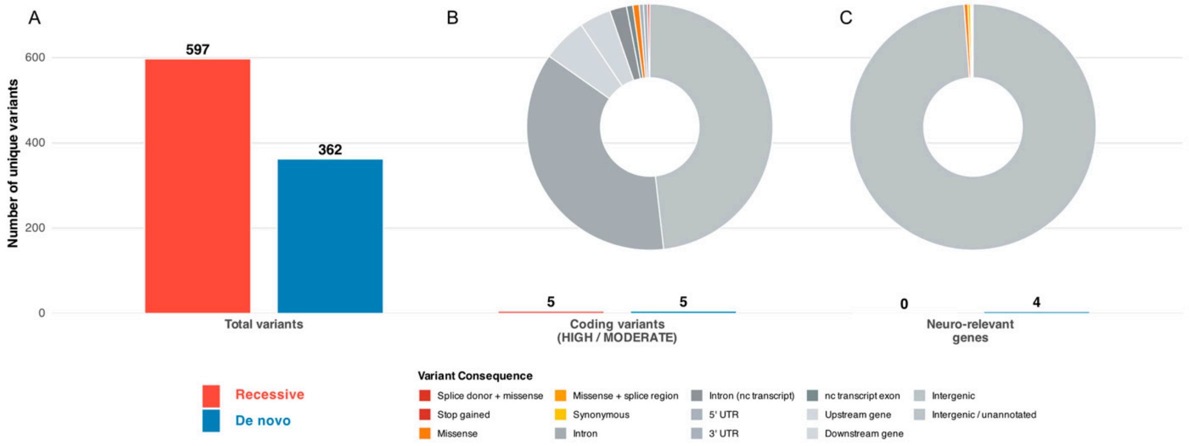

Figure S2. Comparison of de novo/dominant and recessive variant filtering strategies. (A) Number of unique variants retained under each inheritance model. Coding variants were defined as those with HIGH or MODERATE VEP impact. Neuro-relevant genes were identified in neuronal development, synaptic function, or cerebellar biology. **(B-C)** Distribution of variant consequences (VEP annotations, collapsed to one consequence per unique locus) under de novo / dominant filtering (B) and recessive filtering (C), no gene overlapped between the two strategies.

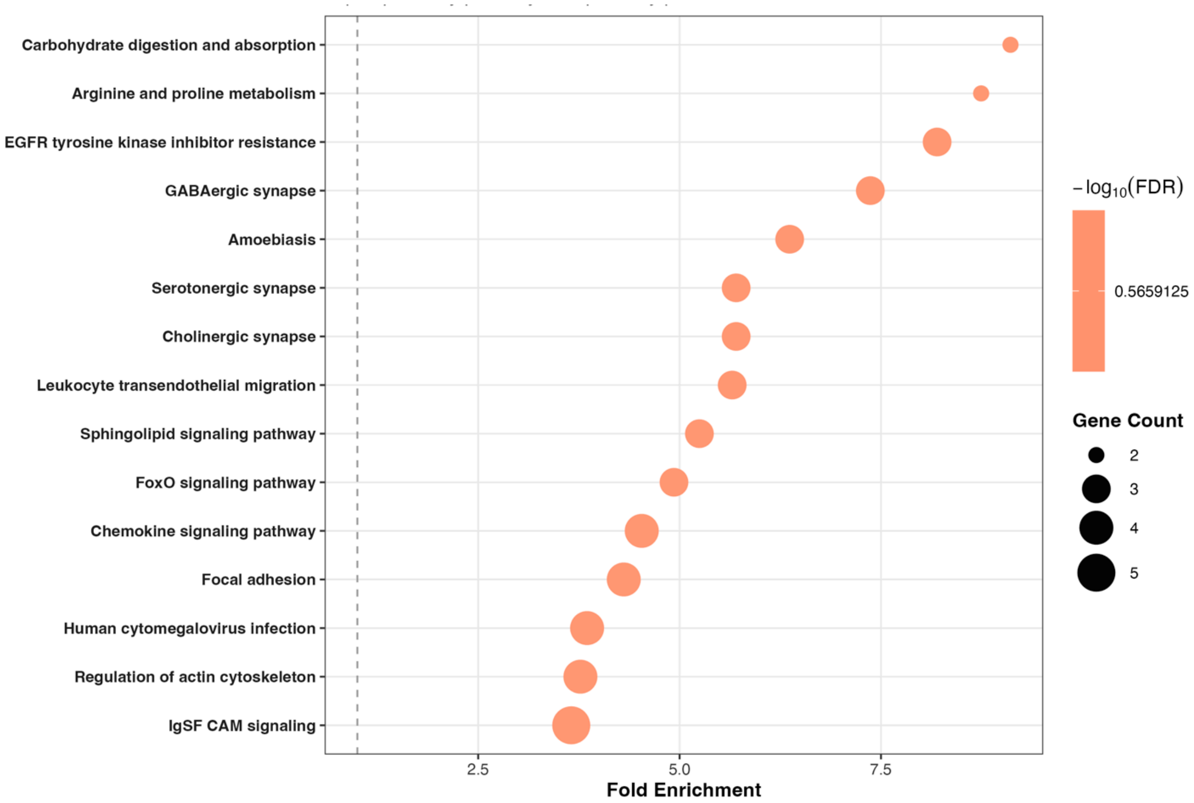

Figure S3. KEGG pathway enrichment of VEP-annotated genes. Canine genes were mapped to one-to-one human orthologues before KEGG analysis. Fold enrichment is shown on the x-axis, bubble size denotes the number of mapped genes, bubble color represents -log10 of the Benjamini-Hochberg false-discovery rate (FDR), and the dashed line marks fold enrichment = 1. The displayed pathways included GABAergic, serotonergic, and cholinergic synapses and regulation of the actin cytoskeleton. However, no pathway remained significant after multiple-testing correction (all displayed terms, FDR = 0.2717). The enrichment pattern is therefore exploratory and does not independently validate PACSIN1 or another candidate gene.

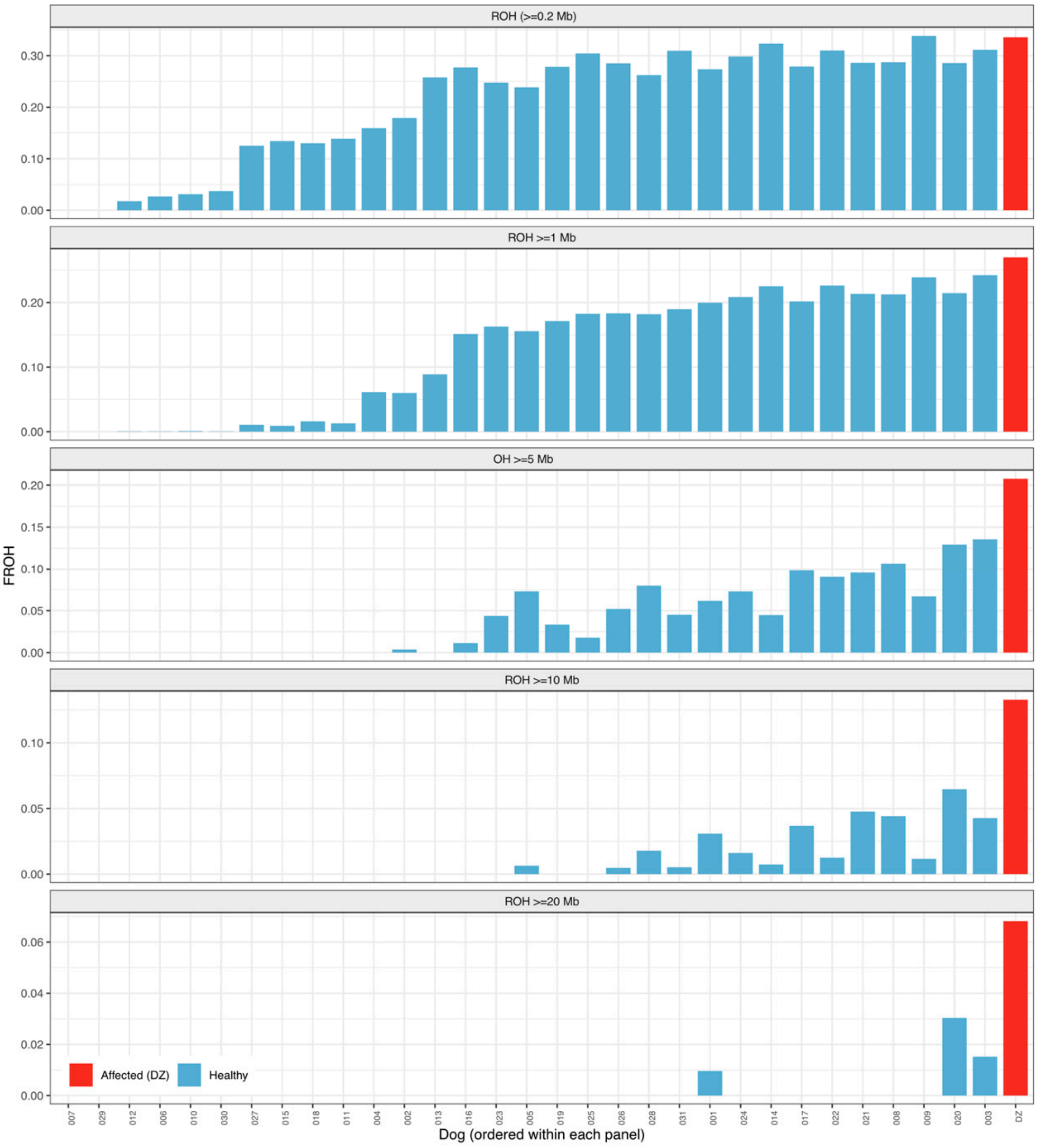

Figure S4. Genome-wide runs of homozygosity burden in DZ and comparison dogs. FROH was calculated as the summed length of autosomal ROH divided by the 2,228.549-Mb callable autosomal span and was evaluated using minimum ROH-length thresholds of 0.2, 1, 5, 10, and 20 Mb. Dogs are ordered independently within each panel; DZ is shown in red. DZ had an overall FROH of 0.3357 (rank 2/32; comparison-dog mean, 0.2076). The excess was strongest for long ROH: for ROH >= 5 Mb, DZ FROH was 0.2076 versus a comparison-dog mean of 0.0407 (5.10-fold; rank 1/32); DZ also ranked first at the 10- and 20-Mb thresholds (FROH = 0.1329 and 0.0682, respectively). This pattern supports elevated recent auto zygosity or shared ancestry in DZ, but the single-case comparison does not establish a causal relationship with the neurological phenotype.

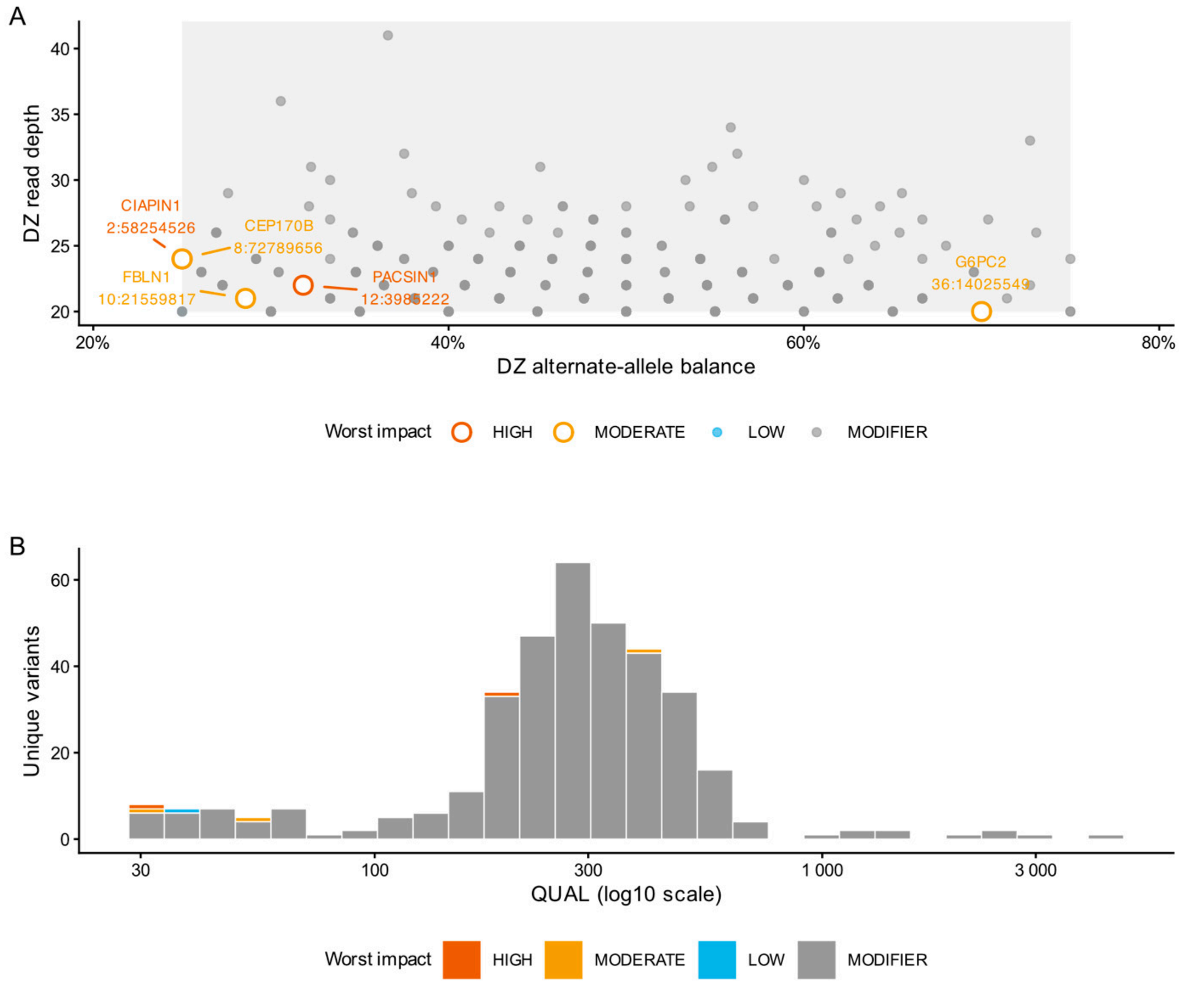

Figure S5. Quality evidence for DZ-specific heterozygous candidate variants. (A) DZ alternate-allele balance plotted against read depth. The shaded region denotes the applied genotype filters (DP >= 20 and alternate-allele balance 0.25-0.75); colors indicate the worst VEP impact, and HIGH or MODERATE candidates are labelled. The *PACSIN1* chr12:3985222 T>G and CIAPIN1 chr2:58254526 variants were classified as HIGH-impact splice-donor candidates. **(B)** Distribution of site-level QUAL values on a log10 scale. The 362 variants comprised 2 HIGH, 3 MODERATE, 1 LOW, and 356 MODIFIER variants. Although DZ genotype metrics passed the specified filters, low site-level depth in some comparison dogs means that reference genotype calls do not prove complete allele absence.
